# Symbolic representations of infinity: the impact of notation and numerical syntax

**DOI:** 10.1007/s00426-024-02050-8

**Published:** 2024-12-02

**Authors:** Ami Feder, Yair Graithzer, Michal Pinhas

**Affiliations:** Department of Psychology, Ariel University, 4070000 Ariel, Israel

## Abstract

Past research indicates that concepts of infinity are not fully understood. In countably infinite sets, infinity is presumed to be perceived as larger than any finite natural number. This study explored whether symbolic representations of infinity are processed as such through contrasts with Arabic and verbal written numbers. Comparisons between the infinity word and number words were responded to faster than comparisons of two number words, but not when the infinity symbol was solely compared to Arabic numbers. Moreover, infinity comparisons yielded distance-like effects, suggesting that infinity (both word and symbol) can be misconceived as a “natural number” closer to larger numbers than small ones. These findings demonstrate difficulty perceiving the physically smallest stimulus (∞) as the upper end-value and seem to reflect a limited understanding of symbolic forms of infinity among adults. They further highlight the impact of notation and numerical syntax on how we process symbolic numerical information.

**Supplementary Information:**

The online version contains supplementary material available at 10.1007/s00426-024-02050-8.

## Introduction

Notions of infinity are interpreted differently in various fields (e.g., mathematics, philosophy, religion), yet they all invoke the highest level of abstract thinking. Attempts to understand infinity exceed our practical, concrete experiences and even our imagination limits. Unlike frequently encountered numbers, infinity is inaccessible and unattainable, and therefore reflects an entirely different concept from numbers. Moreover, concepts of infinity and (natural) numbers differ substantially in the level of abstraction needed to comprehend them. Still, understanding infinity is closely related to understanding concepts such as a set of numbers, the endlessness of numbers, and number systems in general (e.g., Ellis et al., 2018; Falk, 2010; Núñez, 2005; Pinhas, 2024; Rips & Thompson, 2014). Motivated by the common elements in comprehending both infinity and natural numbers, and by the notion that studying unique numerical concepts can reveal fundamental aspects of the way numerical information is conveyed (Pinhas et al., 2021), the present study aimed to illuminate the understanding of how numerical infinity is processed compared to natural numbers, and whether notation and numerical syntax play a role in such processing.

## Comprehending notions of infinity

Numerical infinity has been associated with two different concepts following Aristotle's (384–322 BCE) distinction: *potential infinity*—an unending process, and *actual infinity*—the hypothetical completion of an endless process (Butterworth, 1999; Falk, 2010; Lakoff & Núñez, 2000; Luis et al., 1991; Monaghan, 2001; Núñez, 2005; Stillwell, 2010). In countably infinite sets, counting can be viewed as potential infinity, while the set of natural numbers can be viewed as actual infinity. The understanding of the endlessness of numbers, reflecting the notion of potential infinity, usually develops intuitively in late childhood (Butterworth, 1999; Gelman & Gallistel, 1986). Later on, during adolescence, a conceptualization of actual infinity develops, though in some cases, it may not be reached even in adulthood (e.g., Cheung et al., 2017; Evans, 1983; Falk, 2010; Falk et al., 1986; Gelman, 1980; Hartnett, 1991; Hartnett & Gelman, 1998; Pehkonen et al., 2006). Accordingly, many adults have not advanced beyond a rudimentary comprehension, including undergraduate education students (Kolar & Čadež, 2012) and elementary school teachers (Maria et al., 2010; Singer & Voica, 2003). Consistently, adults demonstrated an impaired understanding of infinity by not ranking the endlessness of numbers as one of the key properties of a possible number system (Rips & Thompson, 2014).

## Symbolic infinity and symbolic number representations

Despite understanding when and how the concept of infinity develops, it is not yet fully understood how comprehension levels of concepts of infinity translate onto symbolic representations of infinity (i.e., ∞ or the infinity word) from the perspective of numerical cognition research. A recent study sought to directly compare the infinity symbol with single and multi-digit numbers, and discovered that while the infinity symbol can be misconceived as a natural number and processed as “larger than” other numbers, it was not associated with the special status of “the largest” (Pinhas, 2024). Hence, it seems that infinity can be placed on the *mental number line*, a numerical magnitude system onto which numbers are mentally ordered along a continuum, spatially oriented from left-to-right (e.g., Dehaene, 1992; Moyer & Landauer, 1967). As a result, the symbolic representation of infinity may be subjected to several robust effects that have been found when numbers are compared in the context of such a representation.

One such effect is the distance effect, referring to faster reaction times (RTs) to comparisons of numbers that are further apart (e.g., 2 vs. 6; distance 4) than closer together (e.g., 2 vs. 3; distance 1) (Moyer & Landauer, 1967). This robust effect occurs in different notations and various number ranges (e.g., Dehaene & Akhavein, 1995; Huber et al., 2016; Moyer & Landauer, 1967; Nuerk et al., 2011). Another common effect is the end effect, which consists of faster RTs when comparing pairs that contain the smallest/largest number in an experimental set than when comparing its mid-range numbers, despite a fixed intrapair distance (Banks, 1977; Moyer & Dumais, 1978). For example, if only single digits are included, comparisons of 1 versus 2 (i.e., the smallest end-value, and a mid-range number, respectively) would be responded to faster than comparisons of 2 versus 3 (i.e., two mid-range numbers). The end effect was also documented in multi-digit number comparisons (Dehaene, 1989; Lozin & Pinhas, 2024; Ratcliff & McKoon, 2020).

A further nuance of the end effect is the two types of end-values: episodic and semantic. Episodic end-values refer to the largest/smallest number within the task-specific set (e.g., Banks, 1977; Leth-Steensen & Marley, 2000). In contrast, semantic end-values are hard-coded in our long-term memory as end-values based on our life experience of “the smallest number,” like 0 or 1 (when 0 is excluded from the set). Hence, only semantic end-values are perceived as such when processing the number values is not part of a task’s requirements (Pinhas & Tzelgov, 2012; Pinhas et al., 2015). Notably, the distance and end effects sometimes interact so that distance effects are attenuated (or even absent) for pairs that contain an end-value (for a possible model, see Leth-Steensen & Marley, 2000).

## Notation effects on number processing

When processing numerical information, notation may affect such processing. Numbers can be represented symbolically in various notations such as verbally (spoken/written words) or using Arabic numbers (digits). This raises the ongoing debate of how numbers are mentally represented in different notation formats—notation-dependent (e.g., Campbell & Epp, 2004; Cohen Kadosh, 2008; Cohen Kadosh & Walsh, 2009; Cohen Kadosh et al., 2008; Myers & Szücs, 2015; Quinlan et al., 2020) or notation-independent (e.g., Dehaene, 1992; Dehaene & Akhavein, 1995; Eger et al., 2003; McCloskey, 1992; Pinel et al., 2001). For instance, the triple-code model (Dehaene, 1992), assumes that number comparison is performed by the analog magnitude code, in which numbers are initially converted into the same abstract “code.” Hence, similar distance effects for comparisons of Arabic and verbal written numbers are predicted. Consistently, Pinel et al. (2001) found that while verbal numbers were compared more slowly than Arabic numbers, similar distance effects were obtained in both notations.

In contrast, Campbell and Epp (2004) postulated that number processing is mediated by notation-specific processes (e.g., words, digits) and not by an abstract code. They further argued that the more familiar and practiced a certain number format is, the more efficient the retrieval of the numerical magnitude information would be. Evidence supporting notation-dependent representation has revealed differences in the distance effects obtained for verbal numbers versus digits. For example, a smaller distance effect was found for verbal numbers than for digits, suggesting a separate comparison mechanism for each notation (Cohen Kadosh, 2008; Cohen Kadosh et al., 2008).

Since past studies provided evidence supporting notation-specific processes, we wished to examine whether different notations of infinity modulate its processing. In other words, when compared to natural numbers, would it matter whether they are compared to the infinity symbol or the infinity word? A difference in notation processing can exhibit the robustness of the infinity concept. It would show us whether the processing of infinity is due to perceptual, or other cognitive factors, or whether it is to do with the concept itself.

## The association between numerical and physical magnitude

Potentially different processing for distinct notations of infinity may be impacted by the inherent association between numerical and physical magnitude. In the Arabic number system, the longer the number string (expressed by the number of digits/places that constitute it), the larger the number’s magnitude. In turn, the number’s length serves as a cue for determining its relative value (e.g., Dixon, 1978; García-Orza et al., 2023; Hinrichs et al., 1982; Huber et al., 2014; Lozin & Pinhas, 2022, 2024; Pinhas, 2024; Varma & Karl, 2013). Number length, together with several other syntactic and lexical features of numbers like digit order and identity, are thought to be encoded via a “visual analyzer” during the early visual analysis of number stimuli (e.g., Cohen & Dehaene, 1991; Dotan & Dehaene, 2020; Dotan & Friedmann, 2018; Dotan et al., 2021). Moreover, identifying number length has been postulated to be a part of the number-to-quantity conversion process (Dotan & Dehaene, 2020).

The association between numerical and physical magnitude is also reflected in verbal number processing. Besides evidence demonstrating that the longer a number word is, the longer it takes to process it, previous studies have reported a word length-value congruity effect (e.g., Kwon & Oh, 2019; Razpurker-Apfeld & Koriat, 2006; Vaid, 1985), which refers to the congruity between number word lengths and their numerical values. In a verbal number comparison task, pairs in which one of the two number words is both longer and numerically larger (e.g., one vs. nine) are categorized as word length-value congruent, and responded to faster, while pairs in which the longer number word is numerically smaller (e.g., three vs. nine) are categorized as word length-value incongruent and responded to more slowly. Hence, the effect is measured as the RT difference between word length-value incongruent and congruent trials and indicates that word length is processed automatically. A similar congruity effect can be computed for mixed-notation comparisons between number words and Arabic numbers.

The word length-value congruity effect resembles the size congruity effect (e.g., Besner & Coltheart, 1979; Henik & Tzelgov, 1982; Paivio, 1975), found typically in a physical size comparison task. In this task, participants encounter pairs of numbers varying in both their numerical values and physical sizes. The instructions explicitly guide participants to disregard the numerical values (considered as the irrelevant dimension) and instead focus on selecting the physically larger/smaller number (deemed the relevant dimension). Generally, responses tend to be more rapid for congruent trials (for example, 2 vs. ), where the numerically larger number aligns with the physically larger size, in contrast to incongruent trials (e.g.,  vs. 4). This effect also reflects the automatic processing of the number values because they are processed albeit being task irrelevant. Such congruity effects, related to the association between numerical and physical magnitude, become relevant when comparing infinity to natural numbers using different notations that present stimuli in varying physical sizes.

Recently, Pinhas (2024) contrasted the infinity symbol (∞) with one-, two-, and three-digit numbers using the numerical and physical comparison tasks. Numerical infinity comparisons did *not* result in upper-end effects (i.e., faster responses for comparisons of infinity and numbers than for number comparisons) but did produce distance-like effects, namely, comparisons of infinity and larger numbers were responded to faster than comparisons of infinity and smaller numbers. In the physical comparison task, comparisons between infinity and single digits yielded a size congruity effect (e.g., faster responses when infinity was physically larger). However, an inverse effect emerged for comparisons of infinity and multi-digit numbers which are physically larger than infinity. Importantly, these findings were replicated when the physical sizes of the stimuli were manipulated or controlled for. Together, Pinhas’s (2024) data showing that the infinity symbol was automatically processed as smaller than multi-digit numbers, demonstrate the strong influence of the physical size of the stimuli and the number of elements comprising them on the perception of numerical concepts as “larger/largest.”

## The present study

The main purpose of the present study was to examine the processing of symbolic representations of numerical infinity in two different notations: the infinity symbol and the Hebrew word for “infinity,” while contrasting them with Arabic and verbal (written) natural numbers. An exploration of the two different notations of infinity expands the study of Pinhas (2024) and can deepen our understanding of processing infinity, and whether it is notation dependent. As infinity is a numerical entity “greater than anything else,” we wondered whether infinity has the status of “*largest*” upper end-value, despite it not being a number representing a concrete quantity. While Pinhas’s (2024) findings did not reveal an upper end effect in comparisons to the infinity symbol, a different pattern may emerge for the infinity word.

It is also of great theoretical interest whether the processing of infinity differs from single and multi-digit numbers, expanding the range of three-digit numbers in Pinhas (2024). To start, single and multi-digit numbers differ in overall length—a perceptual difference associated with numerical value. Notably, the infinity symbol is comprised of only one shape, which orthographically resembles the external representation of single digits, although it is larger than any number. Importantly, in the verbal notation, the representation of infinity includes more than one symbol, like that of other number words. Although number word length also affects verbal number processing (e.g., Kwon & Oh, 2019; Razpurker-Apfeld & Koriat, 2006; Vaid, 1985), the inclusion of verbal notation enables comparing infinity with other numbers, while neutralizing the built-in association between Arabic number length and value. Therefore, we might find unique effects for verbal and Arabic number notation blocks, exemplifying a notation-specific processing, possibly affected by visual features. On the contrary, similar effects in both notations can exemplify the mental processing of infinity as an abstract, notation-independent concept.

To explore these questions, two experiments were conducted, employing the numerical comparison task. In Experiment 1, participants were presented with pairs of single digits or with pairs of infinity and a single digit in different notations: Arabic (e.g., 1 vs. 2; 1 vs. ∞), verbal (e.g., one vs. two; one vs. infinity), or mixed notation (e.g., 1 vs. two; one vs. ∞; 2 vs. infinity). Experiment 2 was identical to Experiment 1 in all aspects aside from the use of multi-digit scale numbers (e.g., 10, 1,000,000,000) instead of single digits.

End and distance effects were used as indicators that infinity was represented as the upper end-value and as a numerical entity mapped onto the numerical magnitude system, respectively (see also Pinhas, 2024; Pinhas et al., 2015; Pinhas & Tzelgov, 2012; Zaks-Ohayon et al., 2021, 2022). If (one or both) symbolic forms of infinity are incorrectly represented on the same ordered mental continuum of concrete magnitudes, as if infinity was a natural number (Ellis et al., 2018), then comparisons between infinity and a number should result in a “distance-like” effect—a linear increase in RTs with increased value of the infinity comparand (i.e., the number compared to infinity; Pinhas, 2024). This would suggest an incorrect perception that larger numbers are closer to infinity. Note that the direction of the “distance-like” effect is opposite to a regular distance effect, consisting of a linear decrease in RTs with increased intrapair distance. In contrast, if comparisons between infinity and numbers do not produce a “distance-like” effect, then the unique properties of infinity (e.g., the distance between any number and infinity is infinite) would seem associated with the perception of one or both of its symbolic representations. In addition, if comparisons between infinity and numbers were to be significantly quicker than comparisons between two numbers, it would suggest that infinity is processed as the “the largest,” corresponding with an upper end-value.

Regarding the influence of notation, because infinity is understood as something larger than anything else and considering the findings of Pinhas (2024) on the infinity symbol and Arabic notation, we expected a distance-like effect in comparisons between infinity and numbers in all notations (Dehaene & Akhavein, 1995; Moyer & Landauer, 1967; Nuerk et al., 2011). As for the end effect, we hypothesized that comparisons to the infinity word would result in an end effect because (a) infinity corresponds to something larger than anything else, and (b) the word “infinity” in Hebrew is longer (i.e., comprised of more letters) than all single-digit number words and half of the multi-digit number words included in Experiments 1 and 2, respectively. This, in turn, makes the infinity word more perceptually distinct and in alignment with the association between numerical and physical magnitude—according to which numerically larger is also physically larger—in most of the examined comparisons to the infinity word.

Noteworthy, in Hebrew, the word infinity (i.e., ), is comprised of a verbal prefix meaning “not,” thus, the word translates to “not-finite.” Relatedly, words or phrases with a negation are known to demand more cognitive resources, slowing down reasoning, and resulting in many errors—a negation effect (e.g., Clark & Chase, 1974; Deutsch et al., 2006; Evans, 1972; Gilbert et al., 1990; Grant et al., 2004; Mayo et al., 2004). In our case, slower responses may be obtained for the infinity word compared to neutral words (like numbers). If “infinity” would indeed be processed as a negation, the presence of other competing processes (e.g., perceiving infinity as the “largest” would fasten responses) may result in similar processing for both numbers and the infinity word. In contrast, weaker )or even absent( end effects were expected in comparisons to the infinity symbol (Pinhas, 2024), particularly when compared to larger Arabic numbers, as the relationship between the numerical and physical sizes of the stimuli is unaligned with the association between numerical and physical magnitude.

## Experiment 1: comparisons of infinity to single-digit numbers

This experiment examined the processing of the infinity symbol and word as contrasted with single-digit numbers in Arabic and verbal notations. Participants performed a numerical comparison task in three different blocked formats. The Arabic number/verbal notation block consisted of comparisons between the infinity symbol/word (in Hebrew) versus a single digit/number word (in Hebrew) and between single digits/number words, respectively. The mixed notation block included comparisons of the infinity symbol versus a number word, the infinity word versus a digit, and a digit versus a number word.

In the Arabic number notation block, the infinity symbol, which represents the largest numeric-like value, was comprised of only one symbol, orthographically similar to single digits. Whereas in the verbal notation block, the word “infinity” in Hebrew () possesses the highest numeric-like value and has the longest word length as well (i.e., comprised of more letters than all of the other single digit number words; Table 1). Therefore, we expected an upper end effect, namely, faster RTs for the infinity word compared to other numbers (verbal or mixed notation blocks), so that infinity would be perceived as the “largest.” If physical size is more salient than value, this effect would be diminished or nullified in the Arabic number notation block (Pinhas, 2024). Note that although the same font size was used for all stimuli, single digits were still always physically smaller (although to a relatively minor extent) than the infinity symbol because of differences in the width and height dimensions of the symbols and given the horizontal orientation of the infinity symbol. This resulted in a confound between the numerical and physical sizes of the stimuli presented (i.e., the infinity symbol was larger both in ‘value’ and in physical size than the single digits) in the Arabic number notation block. However, given that all stimuli presented in the Arabic number notation block were comprised of only one symbol, we expected the physical-numerical size confound to be less prominent compared to the equivalent confound between word length and value in the verbal notation block.

**Table 1:**
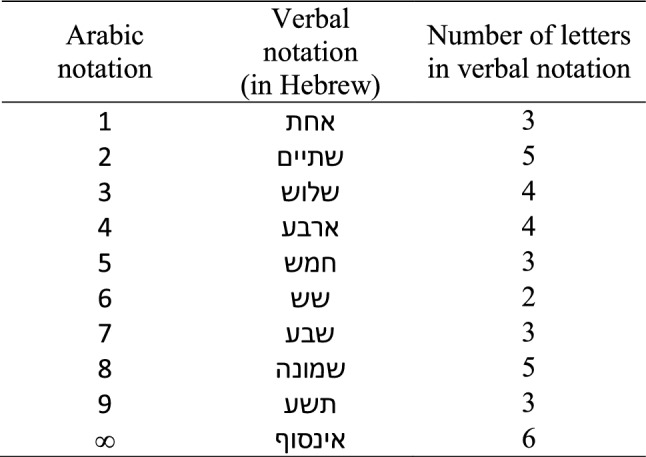
Experiment 1: Detailed stimuli list

The stimuli are presented in the same fonts used during the experiment

Regarding the distance-like effects, we expected the effect to occur in the Arabic number notation block, but not necessarily in the verbal and mixed number notation blocks. Finding such effects will exemplify that the perception of infinity as “larger than” numbers, is not specific to the Arabic notation. Hence, the infinity concept, rather than the symbol or word, is perceived as “larger than” single digits. Lastly, we expected comparisons in Arabic number notation block would yield faster responses than those in verbal and mixed notation blocks (e.g., Cohen Kadosh, 2008; Cohen Kadosh et al., 2008; Dehaene & Akhavein, 1995; Pinel et al., 2001).

### Method

#### Participants

A confirmatory power analysis on 15 participants assumed a Generalized Linear Mixed Model (GLMM) for correct RT, treating the participants as a random variable and block notation, comparison type, and distance as within-participants variables. One-hundred simulations were executed in R (R Core Team, 2022) via the powerSim function of the SIMR package (Green & MacLeod, 2016). Based on the simulated results, the quantity of 24 participants fulfilled prerequisites of α = 0.01 and 95% power for the three-way interaction between block notation, comparison type, and distance.

Twenty-four psychology undergraduate students (mean age = 23.88 years, SD = 1.98; 22 females, 2 males; 5 left-handed; all Hebrew native speakers) from Ariel University participated in the experiment for course credit. The recruitment process excluded participants with dyslexia and/or dyscalculia. Participants provided written informed consent prior to the beginning of the experiment. The study’s protocol was approved by the university’s Institutional Review Board.

#### Apparatus and stimuli

The experiment was conducted online. Stimuli were presented using E-prime® Go software (Psychological Software Tools Inc., Pittsburgh, PA). Participants responded by pressing the “A”/ “L” keys of their keyboard. Stimuli were presented in black on a grey background. Digits and words were presented in 50-point size Calibri font, and the infinity symbol in 50-point size Verdana font. We chose the Verdana font for the infinity symbol because in this font, the infinity symbol looked more similar to the classic symbol than the way it is presented in Calibri font (which is the standard font we use in the lab for number stimuli).

The stimulus set included pairs comprised of numerical values from 1 to 9 presented in Arabic number notation or verbal notation (i.e., Hebrew number words; average word length = 3.8 letters, ranging from 2 to 5 letters), while infinity was presented as a symbol (∞) or a word (in Hebrew; 6 letter word, the longest in the stimulus set). The infinity symbol was physically larger than single digits, and the ratio between the visual areas occupied by the symbols was ~ 1.57 (infinity: single digits). See Table 1 for detailed stimuli.

Two pair types were generated for each comparison block: 1. infinity comparisons – infinity paired with the numbers 1–9, excluding the number 8 due to its physical resemblance to infinity (8 pairs), 2. Single-digit comparisons—each pair included a smaller number (i.e., 1, 2, or 3), and the intrapair distance to the larger number in the pair was 1 to 6 (18 pairs) (for similar designs see Pinhas, 2024; Pinhas et al., 2015; Pinhas & Tzelgov, 2012; Zagury et al., 2022; Zaks-Ohayon et al., 2021, 2022; See Table 2 for example stimuli pairs). Infinity comparisons were evaluated in terms of “the infinity comparand” (i.e., the number compared to infinity; Pinhas, 2024) instead of distance. Moreover, although infinity was compared with the numbers 1–9 (excluding 8), comparisons between infinity and the numbers 7 or 9 were used as “filler pairs” and were not included in the statistical analyses.

**Table 2:**
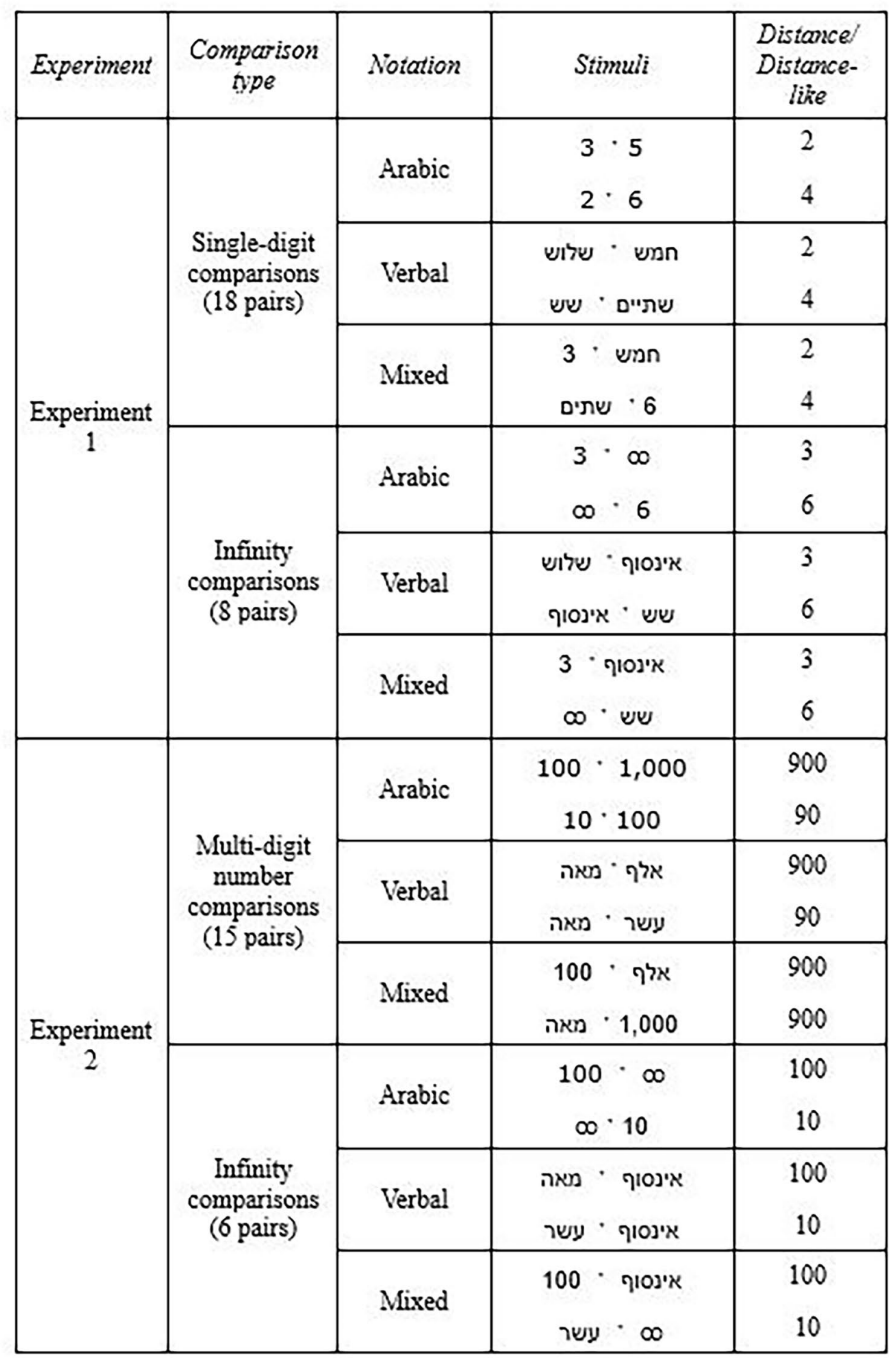
Examples of stimuli pairs in both experiments

The stimuli are presented in the same fonts used during the experiment

Each number within each pair appeared once on the left and once on the right. Each pair appeared 10 times in the Arabic and verbal number notation blocks, giving a total of 520 trials per block [i.e., 26 pairs (8 infinity comparisons + 18 single-digit comparisons) × 2 left/right side × 10 repetitions = 520]. In the mixed notation block, the number of unique pairs was doubled for each comparison type because of the mixed notation combination (e.g., the pair “3 vs. 5” was presented in mixed notation as “3 vs. five” and “three vs. 5”). Hence, each pair was repeated 5 times, producing a total of 520 trials per block [i.e., 46 pairs (16 infinity comparisons + 36 single-digit comparisons) × 2 mixed notation combinations × 2 left/right side × 5 repetitions = 520]. In total, there were 1560 trials in the experiment.

#### Procedure

The experiment was conducted online. Participants received a file of the experiment via email and were instructed to save and open it. The experimenter communicated with the participant via Google Meet™, a video-communication service that enables video chatting and screen sharing. The participants were asked to perform the task in a quiet place and instructed to sit on a chair in front of their computer screen with their index fingers on the response keys. The participants were then guided to start running a short practice phase of the task consisting of 10 example trials, while sharing their computer screen with the experimenter. After the practice, if no problems occurred, the participant continued to the experiment phase without the online presence of the experimenter.

During the experiment, participants were told they would be presented with two stimuli on both sides of the screen that could be a number (in digit or word form) or infinity (in a symbol or word form). They were asked to choose the numerically larger stimulus as quickly and as accurately as possible by pressing the left/right key (“A”/ “L”) if the larger stimulus was presented on the right/left side of the screen, respectively. Each trial started with the presentation of a centered fixation cross for 500 ms, followed by the target stimuli for 500 ms, and then a blank screen for 500 ms. A short self-paced rest break was given after every 65 trials. At each break, feedback of the mean RT and the mean accuracy for the last 65 trials was presented. Trials were randomly ordered, and notation block order was counterbalanced across participants. At the end of the experiment, participants were asked three questions to detect whether their environment during the experiment was conducive to obtaining reasonable RTs. We then used participants’ responses to these three questions as a “filter” for whether any of their RT data should be excluded before performing the statistical analyses (see supplemental materials for more details). The entire online session lasted ~ 45 min.

#### Analysis

Data were analyzed using IBM SPSS (Version 26). The study design and analyses were not pre-registered. Supplemental materials, stimuli, data, and analyses associated with this study are available at https://osf.io/kypsr/

### Results

RTs of correct responses (~ 96% of the data) were initially submitted to a GLMM with block notation (Arabic, verbal, mixed) and comparison type (infinity comparisons, single-digit comparisons) as factors, infinity comparand/distance (1–6) as a covariate, and participants as a random factor. We examined all main effects, as well as the Block Notation × Comparison Type, and the Block Notation × Comparison Type × Infinity Comparand/Distance interactions. Importantly, the meaning of the factor levels for the infinity comparand/distance (i.e., 1–6) differed for each comparison type: they reflected the infinity comparand in infinity comparisons, but the intrapair distance in single-digit comparisons. As a result, distance-like effects in infinity comparisons were expected to result in a linear increase in RT with increased infinity comparand. In contrast, distance effects for single-digit comparisons were expected to emerge as the familiar linear decrease in RT with increased intrapair distance.

A main effect of block notation, χ^2^(2) = 1703.87, *p* < 0.001, confirmed the hypothesis of faster responses in the Arabic (441 ms) than the verbal (656 ms) and mixed notation (618 ms) blocks (both *p* < 0.001). In addition, responses were quicker in the mixed compared to the verbal notation block (*p* < 0.001). Moreover, a main effect of comparison type, χ^2^(1) = 801.47, *p* < 0.001, revealed faster responses for infinity comparisons (535 ms) than for single-digit comparisons (608 ms), consistent with an end effect. Furthermore, there was a main effect for infinity comparand/distance, χ^2^(1) = 13.68, *p* < 0.001, *mean slope* = -12.59, [– 14.82, – 10.36], *p* < 0.001.

**Fig. 1:**
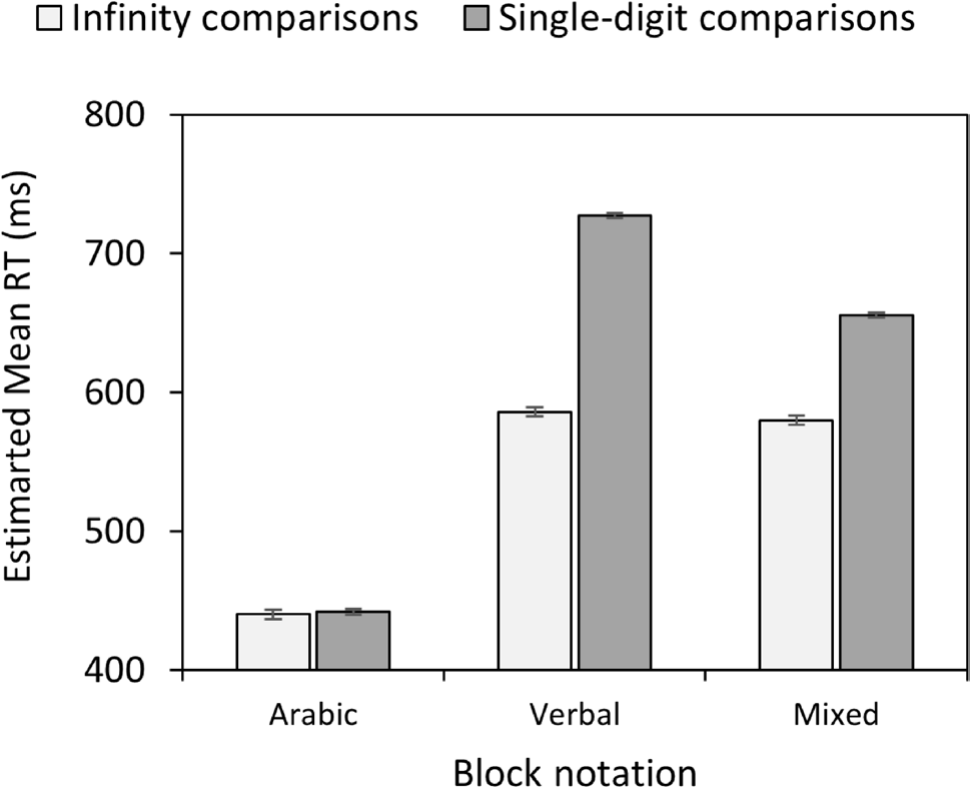
Experiment 1: Estimated mean RT as a function of block notation and comparison type.Vertical bars denote ± standard errors

Moreover, the two-way Block Notation × Comparison Type interaction was significant, χ^2^(2) = 120.10, *p* < 0.001. Importantly, pairwise comparisons revealed faster responses for infinity comparisons than single-digit comparisons—an end effect— in the verbal (*p* < 0.001) and mixed (*p* < 0.001) notation blocks, but not in the Arabic number notation block (*p* = 0.643) (See Fig. 1).

Importantly, the three-way interaction Block Notation × Comparison Type × Infinity Comparand/Distance was also significant, χ^2^(55) = 813.35, *p* < 0.001. To evaluate distance-like/distance effects, we conducted a separate GLMM analysis where both block notation and comparison type variables were split, comparing the linear slopes of the infinity comparand/distance (Table 3). As can be seen in Table 3, all slopes were significant. In infinity comparisons, all slopes were positive, indicating slower reactions with increased infinity comparand, while in single-digit comparisons, all slopes were negative, indicating faster reactions with increased distance.

**Table 3:** Experiment 1: Mean slopes as a function of block notation, comparison type, and infinity comparand in infinity comparisons/distance in single-digit comparisons

*Block notation*	*Comparison type*	*Mean slope*	*[95% Wald CI]*	*p*
Arabic	Infinity comparisons	11.04	[8.57, 13.53]	< .001
	Single-digit comparisons	– 8.07	[– 9.43, – 6.71]	< .001
Verbal	Infinity comparisons	4.11	[0.44, 7.79]	.028
	Single-digit comparisons	– 12.59	[– 15.28, – 9.90]	< .001
Mixed	Infinity comparisons	7.44	[3.74, 11.14]	< .001
	Single-digit comparisons	– 16.26	[– 18.96, – 13.57]	< .001

A significant positive/negative mean slope is consistent with a distance-like/distance effect, respectively

Next, we further inspected performance in the mixed notation block by differentiating between three comparison types: (a) infinity symbol versus a number word, (b) infinity word versus a number, and (c) a number versus a number word. Our purpose was to test the significance of the end and distance-like/distance effects in this block for the two types of infinity comparisons. First, a GLMM analysis revealed a main effect of comparison type, χ^2^(2) = 271.52, *p* < 0.001, demonstrating significant end effects for each of the two types of infinity comparisons: comparisons of the infinity symbol versus a number word (548 ms) were responded to faster than comparisons of a number versus a number word (655 ms), *p* < 0.001, and comparisons of the infinity word versus a number (612 ms) were also responded to faster than comparisons of a digit versus a number word (*p* < 0.001). Second, an additional GLMM analysis revealed a significant Comparison Type × Infinity Comparand/Distance interaction, χ^2^(3) = 165.93, *p* < 0.001. To evaluate distance-like/distance effects, we conducted a separate GLMM analysis where comparison type was split, comparing the linear slopes of the infinity comparand/distance. Significant distance-like effects emerged for both types of infinity comparisons (comparisons of the infinity symbol versus a number word, *mean slope* = 6.66, [1.29, 112.02], *p* = 0.015; comparisons of the infinity word versus a digit, *mean slope* = 8.32, [3.41, 13.22], *p* = 0.001), as well as a distance effect for a digit versus a number (*mean slope* = -16.26, [– 18.96, – 13.57], *p* < 0.001).

Additional analyses examined the influence of word length on verbal number processing in the verbal and mixed notation blocks (See supplemental materials).

### Discussion

The findings of Experiment 1 revealed that infinity comparisons yielded distance-like effects in all notation blocks. Moreover, they revealed faster RTs for infinity comparisons compared to single-digit comparisons in the verbal and mixed notation blocks, demonstrating an end-effect for infinity in these blocks. However, and consistent with Pinhas (2024), this end effect was not significant in the Arabic number notation block. Interestingly, in the mixed notation block, although comparisons to *both* symbolic forms of infinity produced faster responses than number comparisons, producing significant end effects, comparisons between the infinity word and Arabic numbers were the fastest (e.g., infinity vs. 6). Therefore, it seems that infinity was perceived as an end-value only when the word “infinity” was included in the block, but not when infinity was solely presented as a symbol. Furthermore, our findings of an end effect for the infinity word which contains a negation prefix are inconsistent with a negation effect (e.g., Clark & Chase, 1974; Deutsch et al., 2006; Evans, 1972; Gilbert et al., 1990; Grant et al., 2004; Mayo et al., 2004), where negation prefixes are usually processed slower. Together, the infinity word and symbol seem to have been incorrectly mapped onto the numerical magnitude system, as if each was a natural number representing a concrete quantity.

Importantly, while a plausible interpretation of the distance-like effect is that both symbolic forms of infinity were perceived as “larger than” numbers, only the infinity word may be perceived as the upper end-value or “the largest” (evidenced by faster RTs for infinity comparisons than single-digit comparisons), indicating that notation can have a crucial influence on the way numerical information is conveyed (e.g., Campbell & Epp, 2004; Cohen Kadosh, 2008; Cohen Kadosh & Walsh, 2009; Cohen Kadosh et al., 2008; Myers & Szücs, 2015; Quinlan et al., 2020). Moreover, while RTs of infinity comparisons were fastest for the Arabic number notation block, and although there was a distance-like effect in this notation, participants did not process the infinity symbol as the “largest” (see also Pinhas, 2024).

What might account for the differential processing of the infinity word and symbol? One possibility may be that the infinity symbol is not familiar enough, and therefore not strongly associated with the meaning of infinity. Alternatively, the infinity symbol, being comprised of one shape, orthographically resembles single-digit numbers that represent small quantities. Considering the syntactical association between numerical and physical magnitude, a single shape seems less suitable for denoting the largest value (Pinhas, 2024). In contrast, the word “infinity” in Hebrew is indeed longer (i.e., comprised of more letters) than all single-digit number words in Hebrew. Accordingly, the influence of word length in the verbal notation (e.g., Kwon & Oh, 2019; Razpurker-Apfeld & Koriat, 2006; Vaid, 1985) may have significantly contributed to perceiving the infinity word as “the largest” in the verbal and mixed notation blocks, as was evidenced in our findings (See supplemental materials).

Experiment 2 attempted to replicate and extend these findings by including comparisons between infinity and multi-digit numbers. In turn, this also enabled us to examine the processing of the infinity word when it was not the longest word in the set.

## Experiment 2: comparisons of infinity to multi-digit numbers

In Experiment 2, as in Experiment 1, we compared infinity with numbers in Arabic, verbal, and mixed notation blocks. However, in this experiment, we utilized only multi-digit numbers. All numbers were single scale number words ranging from ten to a trillion, thereby including both “small” and “large” multi-digit numbers. Importantly, in this stimulus set, infinity (6 letters long in Hebrew) was not the longest word in the set, like in the verbal block of Experiment 1. Instead, the Hebrew number words “billion” and “trillion” are longer (both 7 letters long). Furthermore, the word “million” is the same letter length as the word “infinity” (Table 4). Thus, in one third of the verbal infinity comparisons, the infinity word was shorter than the number word it was compared with, in one sixth of the verbal comparisons it was of identical length, and in the remaining half the infinity word was longer. Also, in contrast to the Arabic number notation block of Experiment 1, in which the infinity symbol was slightly physically larger than the single digits (despite using the same font size), in the Arabic number notation block of Experiment 2, which included only multi-digit numbers, the infinity symbol was comparatively the largest numerically but the smallest physically (i.e., comprised of only one shape).

**Table 4:**
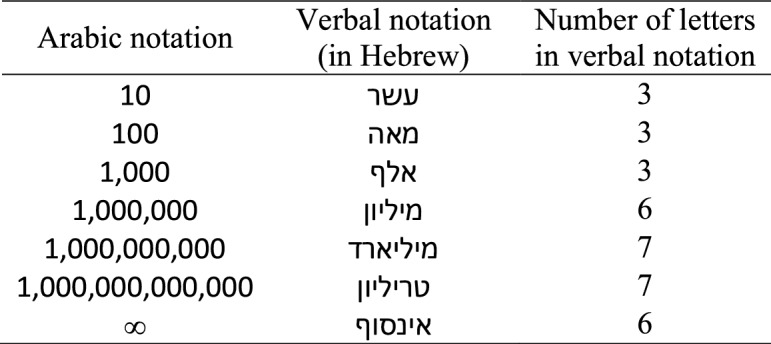
Experiment 2: Detailed stimuli list

The stimuli are presented in the same fonts used during the experiment

Accordingly, we expected to replicate the findings of Experiment 1 showing faster reactions for infinity comparisons in the verbal and mixed notation blocks, consistent with the end effect. Notably, the fact that infinity was the physically smallest symbol in the Arabic number notation block of Experiment 2, goes strongly against the syntactic association of “physically smaller means numerically smaller,” still, it makes the infinity symbol perceptually prominent (considering it is the only one-shape symbol in the block). Such perceptual uniqueness may result in an end effect after all. Further, like in Experiment 1, we expected distance and distance-like effects to emerge for number comparisons in all notation blocks. Finally, following Experiment 1 and previous studies’ (e.g., Cohen Kadosh, 2008; Cohen Kadosh et al., 2008; Dehaene & Akhavein, 1995; Pinel et al., 2001) findings, we expected faster responses in the Arabic number notation block compared to the verbal and mixed notation blocks.

### Method

#### Participants

Twenty-four psychology undergraduate students (mean age = 23.83 years, SD = 1.69; 21 females, 3 males; 4 left-handed; all Hebrew native speakers) from Ariel University participated in the experiment for course credit.

#### Apparatus and stimuli

The stimulus set included the infinity symbol, the infinity word (in Hebrew, 6 letters long), and the six scale numbers from ten to a trillion (ten, hundred, thousand, million, billion, and trillion) in both Arabic number and verbal (i.e., Hebrew number words; average word length = 4.83 letters, ranging from 3 to 7 letters) notations (See Table 4 for detailed stimuli). In each one of the notation blocks, the pairs were presented in all possible permutations and each pair appeared 8 times, giving a total of 336 trials for the Arabic number and verbal notation blocks [i.e., 21 pairs (6 infinity comparisons + 15 multi-digit number comparisons) × 2 left/right side × 8 repetitions = 336]. In the mixed notation block, each pair was repeated 4 times (instead of 8) given the doubled number of unique pairs due to the mixed notation combinations [i.e., 42 pairs (12 infinity comparisons + 30 multi-digit number comparisons) × 2 left/right side × 4 repetitions = 336]. Hence, the experiment included 1,008 trials in total.

#### Procedure

The procedure was the same as Experiment 1, with the exception that (a) a short rest break was given after every 56 trials throughout the experiment, and (b) the whole online session lasted ~ 35 min.

All other details of the methodology were identical to that of Experiment 1.

### Results

RTs of correct responses (~ 96% of the data) were initially submitted to a GLMM analysis with block notation (Arabic, verbal, mixed) and comparison type (infinity comparisons, multi-digit number comparisons) as factors, log values of the infinity comparand/distance as a covariate, and participants as a random factor. As in Experiment 1, the model included all main effects, and the Block Notation × Comparison Type and Block Notation × Comparison Type × log Infinity Comparand/Distance interactions. A main effect of block notation, χ^2^(2) = 1,004.76, *p* < 0.001, reflected faster responses in the Arabic number notation block (505 ms) compared to the verbal (719 ms), *p* < 0.001, and mixed (708) notation blocks, *p* < 0.001. There was also a significant difference between the mixed and verbal notation blocks, *p* = 0.012. Furthermore, a main effect of comparison type, χ^2^(1) = 1,228.24, *p* < 0.001, reflected faster responses for infinity comparisons (614 ms) than for multi-digit number comparisons (674 ms)—an end effect. There was no main effect for log infinity comparand/distance, χ^2^(1) = 0.689, *p* = 0.406.

**Fig. 2:**
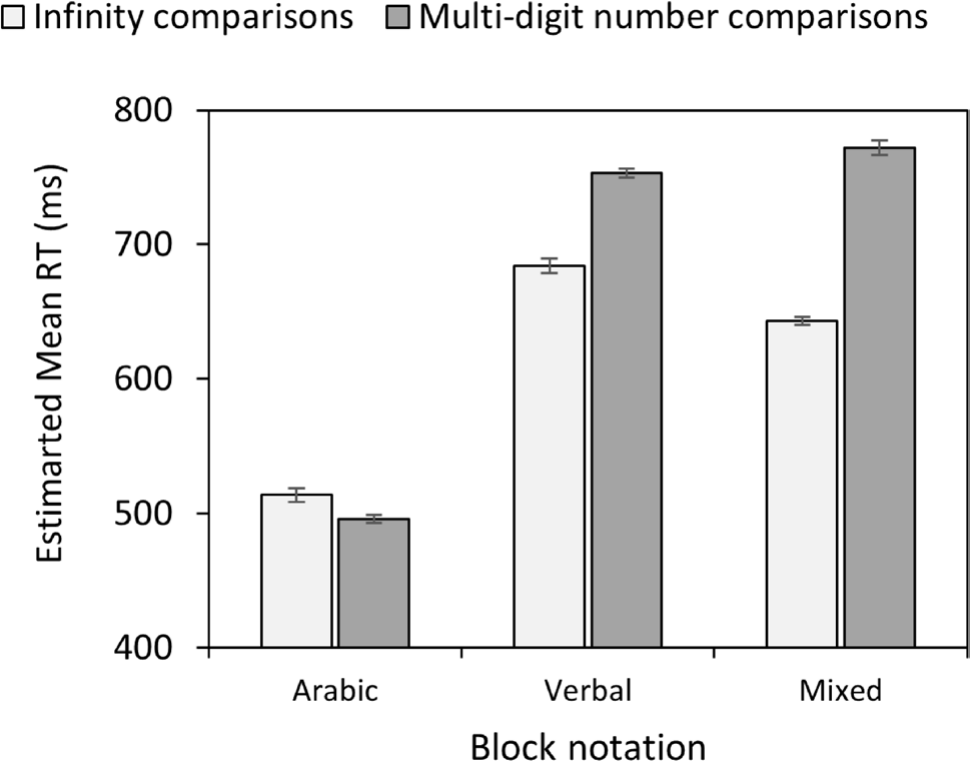
Experiment 2: Estimated mean RT as a function of block notation and comparison type. Vertical bars denote ± standard errors

In addition, the Block Notation × Comparison type interaction was significant, χ^2^(2) = 435.65, *p* < 0.001. Pairwise comparisons revealed significantly faster reactions for infinity comparisons compared to multi-digit number comparisons in the mixed (*p* < 0.001) and verbal (*p* < 0.001) number notation, that is, an end effect. Moreover, unlike Experiment 1, there was a reversed end effect in the Arabic notation, albeit much smaller than the two other notations (Arabic, *p* = 0.005, See Fig. 2).

Importantly, the Block Notation × Comparison Type × log Infinity Comparand/Distance interaction was also significant. To evaluate distance-like/distance effects, we conducted a separate GLMM analysis where both block notation and comparison type variables were split, comparing the linear slopes of the log infinity comparand/distance (Table 5). As Table 5 shows, all slopes were significant. For infinity comparisons, all slopes were positive, indicating slower reactions with increased log infinity comparand. In contrast, for multi-digit number comparisons, all slopes were negative, indicating faster reactions with increased log distance.

**Table 5:** Experiment 2: Mean slopes as a function of block notation, comparison type, and log infinity comparand in infinity comparisons/log distance in multi-digit number comparisons

Block notation	Comparison type	Mean slope	[95% Wald CI]	p
Arabic	Infinity comparisons	4.38	[3.05, 5.71]	< .001
	Multi-digit number comparisons	– 4.10	[– 5.19, – 3.01]	< .001
Verbal	Infinity comparisons	19.37	[17.17, 21.56]	< .001
	Multi-digit number comparisons	– 20.56	[– 22.59, – 18.54]	< .001
Mixed	Infinity comparisons	8.72	[6.70, 10.74]	< .001
	Multi-digit number comparisons	– 9.96	[– 12.17, – 7.76]	< .001

A significant positive/negative mean slope is consistent with a distance-like/distance effect, respectively

Next, as in Experiment 1, we further examined performance in the mixed notation block to test the significance of the end and distance-like/distance effects for the two types of infinity comparisons. Hence, we differentiated between three comparison types (a) infinity symbol versus a number word, (b) infinity word versus a number, and (c) a number versus a number word. First, a GLMM analysis revealed a main effect of comparison type, χ^2^(2) = 430.39, *p* < 0.001, demonstrating significant end effects for each of the two types of infinity comparisons: comparisons of the infinity symbol versus a number word (587 ms) were responded to faster than comparisons of a number versus a number word (772 ms), *p* < 0.001, and comparisons of the infinity word versus a number (708 ms) were also responded to faster than comparisons of a digit versus a number word (*p* < 0.001). Second, additional GLMM analysis revealed a significant Comparison Type × log Infinity Comparand/Distance interaction, χ^2^(3) = 153.24, *p* < 0.001. To evaluate distance-like/distance effects, we conducted a separate GLMM analysis where comparison type was split, comparing the linear slopes of the log infinity comparand/distance. Significant distance-like effects were obtained for both types of infinity comparisons (comparisons of the infinity symbol versus a number word, *mean slope* = 4.03, [1.77, 6.28], *p* < 0.001; comparisons of the infinity word versus a number, *mean slope* = 15.09, [11.87, 18.31], *p* < *0.0*01), as well as a distance effect for a number versus a number word, *mean slope* = -9.96, [– 12.17, – 7.76], *p* < *0.0*01).

Additional analyses examined the influence of word length on verbal number processing in the verbal and mixed notation blocks (See supplemental materials).

### Discussion

In Experiment 2, we investigated infinity comparisons with multi-digit numbers to replicate and extend the findings of Experiment 1, which compared infinity with single digits. In both experiments, there was a significant distance-like effect in all notations. These findings of distance-like effects seemingly imply an incorrect mapping onto the numerical magnitude system of both symbolic forms of infinity, as if each represented a natural number that is numerically “larger than” other natural numbers.

Moreover, in Experiment 2, infinity comparisons were responded to faster than number comparisons in the verbal and mixed notation blocks, demonstrating end effects. In negation to the Arabic notation block of Experiment 1, where there was no end effect in infinity comparisons to single digits, in Experiment 2, there was a significant *reversed* end effect in the Arabic notation block. Although this reversed end effect was much smaller than the (regular) end effects obtained in the other blocks, it indicates that multi-digit number comparisons were responded to significantly *faster* than infinity comparisons. Notably, the infinity symbol was perceptually unique in the Arabic number notation block of Experiment 2 because it was compared with multi-digit numbers. Hence, it was the only single-shaped symbol. Such perceptual uniqueness stresses the incongruity between the orthography of the infinity symbol and numerical syntax (i.e., physically smaller is associated with numerically smaller). Therefore, it seems that participants associated longer with larger (e.g., Dixon, 1978; García-Orza et al., 2023; Hinrichs et al., 1982; Huber et al., 2014; Lozin & Pinhas, 2022, 2024; Pinhas, 2024; Varma & Karl, 2013) even though infinity is infinitely larger than any (multi-digit) number. This result is consistent with Pinhas (2024), showing a reversed size congruity effect for physical comparisons of infinity and multi-digit numbers, even when the physical sizes of the stimuli were manipulated or controlled for. These findings emphasize the role of the number’s overall length and the amount of digits of which it comprised in the process of evaluating numerical magnitude (Dotan & Dehaene, 2020; Pinhas, 2024).

Furthermore, although in the verbal infinity comparisons of Experiment 2 the infinity word was longer than the number word it was compared with only in half of the trials, we found a significant influence of word length on participants’ responses (See supplemental materials, e.g., Kwon & Oh, 2019; Razpurker-Apfeld & Koriat, 2006; Vaid, 1985). Therefore, like in Experiment 1, word length seems to have contributed to the perception of the infinity word as “the largest” in the verbal and mixed notation blocks.

Collectively, the results of Experiment 2 seem to reinforce the notion that the processing of symbolic forms of infinity is dependent on notation, and whether the notation is consistent with the association between numerical and physical magnitude that characterizes numerical syntax.

## General discussion

The primary goal of the present study was to explore the processing of infinity—both symbol and word (in the Hebrew language). We examined comparisons between infinity and numbers, as well as number comparisons, in three different notation blocks: Arabic numbers, verbal (written) numbers, and mixed. In Experiment 1, the numbers were single digits, whereas in Experiment 2, they were multi-digit numbers. We designed the experiments to address the question of whether infinity is perceived as “the largest,” or the upper end-value, when compared with natural numbers. We also aimed to examine whether processing infinity is influenced by its notation. The results suggest that processing infinity as “the largest” was reliant upon notation and numerical syntax. Our data also suggest a limited understanding of symbolic forms of infinity, as it may be incorrectly perceived as a member of the numerical magnitude system, as a “natural number.”

It can be claimed that our design nudged participants to perceive infinity as part of the numerical magnitude system because we asked them to compare infinity to other numbers. If this is the case, the results might not exhibit a limited understanding of infinity, but rather that participants can flexibility, and by task demand, assign infinity to the numerical magnitude system. However, recent findings from physical size comparisons tasks of infinity symbol and Arabic numbers reveal there may be an automatic processing of infinity as “a number,” namely, mapping the infinity symbol onto the numerical magnitude system even when its ordinal status is task-irrelevant and the correct answer in infinity comparisons varies (Pinhas, 2024). Furthermore, the reversed end effect for infinity comparisons in the Arabic notation block of Experiment 2 suggests that perceptual features play a factor in processing infinity. However, if perceptual processing would have exclusively guided infinity comparisons, or if infinity stood out only based on its negation prefix, then only end effects, but not distance-like effects, would have been expected for such comparisons, which was not the case here, or in Pinhas (2024).

### The end effect in infinity comparisons

The impact of notation on processing infinity was evidenced by the end effect (e.g., Banks, 1977; Dehaene, 1989; Leth-Steensen & Marley, 2000; Lozin & Pinhas, 2024; Moyer & Dumais, 1978; Ratcliff & McKoon, 2020) we obtained in the verbal and mixed notation blocks, but not in the Arabic one of Experiment 1, consistent with Pinhas (2024). However, in Experiment 2, there was a smaller *reversed* end effect for infinity comparisons in the Arabic notation block. Such finding of a reversed and weaker end effect for infinity comparisons in the Arabic compared to the other notation blocks are plausibly explained by the difficulty in depicting “the largest” numerical entity as a single symbol, which runs contrary to the built-in association between the numerical and physical magnitude of the decimal structure (Pinhas, 2024), according to which “numerically larger” corresponds with “physically larger,” namely, a longer string of symbols. Consequently, it seems harder to reconcile the infinity symbol’s small physical size with it being “the largest” numerical entity. Moreover, this conclusion is also in line with the recent findings by Pinhas (2024), where physical size comparison tasks demonstrated faster reactions for comparisons of single digits with the infinity symbol when infinity was physically larger, but slower reactions when the infinity symbol was compared to multi-digit numbers, and thus, physically smaller.

More generally, there is a small amount of reported upper end effects for multi-digit numbers in Arabic notation. One study discovered a small upper end effect (and an equivalent small lower end effect; Verguts et al., 2005) after training a neural network model using parity judgments of the numbers 1–15. Furthermore, Dehaene (1989) discovered symmetric upper and lower end effects for numbers between 20 and 99. As opposed to this, Ratcliff and McKoon (2020) did not find evidence of (lower/upper) end effects when asking participants to arrange dot arrays ranging from 11 to 90 on an arc or numbers on a line flanked by 1 to 100. Importantly, these examples (except for Verguts et al., 2005) display an end effect for same-scale comparisons. Therefore, they are similar to Experiment 1, where orthographically, both infinity and single-digit numbers in the Arabic notation are comprised of a single symbol. In Experiment 2, the Arabic notation block contained numbers from different-scales and infinity as the only single-shaped symbol, and we discovered a reversed end effect. Such findings are consistent with a recent study (Lozin & Pinhas, 2024) reporting both lower and upper end effects for different-scale number comparisons, using a wide range of end-values from 1 to 9,000,000.

Moreover, and as suggested by Pinhas (2024), the null or reversed end effects obtained for infinity comparisons in the Arabic number notation block possibly indicate the infinity symbol was processed as a “small” or at least “not the largest” numerical entity based on a crude evaluation of its numerical size as derived from the physical characteristics of its symbol. Consistently, a mental “visual analyzer” mechanism that was suggested to encode the overall number length extracts this feature during the early visual analysis of multi-digit number stimuli (e.g., Cohen & Dehaene, 1991; Dotan & Dehaene, 2020; Dotan & Friedmann, 2018; Dotan et al., 2021) and use this information as part of the number-to-quantity conversion process (Dotan & Dehaene, 2020). Such a visual analyzer mechanism may also encode the length of numerical symbols that are not numbers, like infinity. Additionally, an early automatic processing of syntactical features may “override” subsequent refined processing of numerical value (for a possible model, see Lozin & Pinhas, 2022).

In contrast, the enlarged end effects found among infinity comparisons in the verbal and mixed notation blocks suggest that infinity was more easily processed as “the largest” or the upper end-value under these conditions. In the verbal notation blocks, the processing of the infinity word as “the largest” was presumably affected by word length-value congruity, capturing the association between word length and numerical value (e.g., Kwon & Oh, 2019; Razpurker-Apfeld & Koriat, 2006; Vaid, 1985, See supplementary materials for these analyses). Akin, the infinity word was indeed the longest word in Experiment 1, and the longest word in half of the trials in Experiment 2; therefore, it may have been more easily processed as an upper end-value. In the mixed notation block of Experiment 1, comparisons between the infinity word and Arabic single digits (“stimulus length-value congruent”) were responded to faster than comparisons between the infinity symbol and verbal single digits (“stimulus length-value incongruent”), consistent with the stimulus length-value congruity effect. Thus, although both forms of infinity have seemed to be processed as an upper end-value, the infinity word was processed as such to a greater extent, likely due to the greater length of the word than the symbol.

Taken together, the current findings of stronger end effects for infinity comparisons in the verbal and mixed notation blocks, and null or reversed end effects in the Arabic number notation block, suggest that symbolic forms of infinity are not perceived as upper semantic end-values (like 0 and 1 for semantic lower end-values; Pinhas et al., 2015; Pinhas & Tzelgov, 2012). Namely, symbolic forms of infinity do not seem to be hard-wired in long-term memory as “the upper end-value” or “the largest” based on prior experience. Instead, processing symbolic forms of infinity as “the largest” may reflect an episodic organization of the ordinal relations of the set’s stimuli, guided by notation and numerical syntax.

### The distance-like effect in infinity comparisons

In contrast to the findings of the end effect, distance-like effects in infinity comparisons did not seem to be impacted by notation. In all cases, distance-like effects emerged for comparisons between both symbolic forms of infinity and numbers, implying an incorrect perception that larger numbers are closer to infinity than smaller numbers. Hence, these findings suggest a limited understanding of numerical infinity, and are aligned with findings of a previous study directly testing comparisons of infinity symbol and Arabic numbers (Pinhas, 2024), as well as other studies showing a general incomprehension of notions of infinity by adults (Falk, 2010; Kolar & Čadež, 2012; Maria et al., 2010; Pehkonen et al., 2006), particularly of actual infinity (Falk, 2010; Rips & Thompson, 2014), notwithstanding their use of different methodologies (e.g., questionnaires, interviews) and lack of inspecting symbolic forms of infinity.

**Notation-dependence of symbolic forms of infinity.**

More broadly, our data showing differential processing for the infinity symbol and word are consistent with notation-dependent theories postulating that number processing is mediated by notation-specific processes and not by an abstract code (e.g., Campbell & Epp, 2004; Cohen Kadosh, 2008; Cohen Kadosh & Walsh, 2009; Cohen Kadosh et al., 2008; Myers & Szücs, 2015; Quinlan et al., 2020). If the representation of infinity was indeed notation-independent, as suggested regarding numbers by some researchers (e.g., Dehaene, 1992; Dehaene & Akhavein, 1995; Eger et al., 2003; McCloskey, 1992; Pinel et al., 2001), we would have expected similar processing of both symbolic forms of infinity in all notation blocks. Accordingly, our findings extend the notion of notation-dependency from numbers to the infinity concept. Moreover, our evidence of notation-dependency for infinity symbol may be another reflection of a rather weak understanding of numerical infinity combined with robust comprehension of natural numbers, given that such notation-dependency was not apparent in the present study for number comparisons. Furthermore, the primary influence of the notation type, and the perceptual characteristics of the stimuli conveyed by it, may be a result of “referential processing.” According to Patel and Varma (2018), task- and stimulus-specific representations are utilized while processing abstract numerical concepts, such as infinity, in this case; thus, when symbolic forms of infinity are compared to numbers, the stimuli’s physical sizes probably serve as main referents.

Our results strongly suggest that to increase the probability of perceiving infinity as “the largest,” its symbolic form might require better alignment with the association between numerical and physical magnitude conveyed by the syntax of the Arabic number system (see also Pinhas, 2024). Hence, the larger the numerical value, the more symbols should comprise it and the longer it should be. Together with other studies demonstrating the challenges involved in various form of syntactic processing of numbers among children and adults (Cheung & Ansari, 2021; Moura et al., 2013; Power & Dal Martello, 1990, 1997; Steiner et al., 2021), the present findings showcase the central role of syntax in processing symbolic numerical information.

### Study limitations and future directions

Despite the contribution of the current findings, there are some limitations. First, for consistency’s sake with past research (Pinhas, 2024), we did not explicitly measure the participants’ knowledge regarding the infinity concept. Still, asking the participants directly about their understanding of infinity in addition to examining comparisons to symbolic forms of infinity could have provided further insight to the concept and its perception. Therefore, future research can examine possible correlations between participants’ behavioral performance and declared knowledge of infinity. A second limitation concerns differences in stimuli familiarity. Accordingly, comparing numbers, particularly single digits, with the infinity symbol, may not have only been a comparison between exact numbers and an abstract concept, but also a comparison between very familiar objects and a less familiar one, respectively. Third, while the present study focused on contrasting two symbolic forms of infinity in various notation and number range contexts, we did not compare infinity with other upper end-values. Such comparisons might be revealing concerning the underlying representations of concepts of infinity and await future investigation.

Moreover, the current findings generate new questions concerning symbolic forms of infinity that call for further examination. One question is whether comparisons between infinity and non-scale multi-digit numbers would produce similar results as those to scale numbers; knowing the answer to this question would enable a better understanding of how infinity is processed concerning “large” numbers. Additional questions arise from our findings of end effects which suggest a notation-specific, *lingual* effect for infinity: it was more easily perceived as an upper end-value when appearing in word form compared to when appearing (solely) as a symbol. Future studies may aim to identify the precise factor(s) that created this effect; whether it originates from word length, distinctiveness, or frequency, and whether it is phonological, morphological, orthographical, or relates to putative semantic organization of the number word lexicon. Relatedly, following studies revealing possible language markedness in multi-digit number comparisons (e.g., Bahnmueller et al., 2015, 2020; Moeller et al., 2015; Nuerk et al., 2005), it may be that the specific language used here for the stimuli (Hebrew) affected infinity processing, as well as single- and multi-digit numbers. Therefore, the inclusion of other languages in future research may help shed light on the effect of language on processing infinity (Kim et al., 2012).

## Conclusions

The present study expands our understanding of how we process symbolic forms of infinity—symbol or word—in relation to numbers in Arabic, verbal, and mixed notations. Our novel findings suggest that the processing of infinity as the upper end-value or “the largest” was dependent on both notation type and numerical syntax. It was harder to perceive the infinity symbol compared to word as an upper end-value because the symbol is inconsistent with the association between numerical and physical magnitude conveyed by the syntactic rule “numerically larger is also physically larger.” The current findings also show that both the infinity symbol and word can be misconceived as a natural number, incorrectly mapped onto the numerical magnitude system. Hence, consistent with past studies, our results suggest a rather poor understanding of numerical infinity among adults. The fact that the processing of infinity was impacted by its external symbolization, unlike comparisons between numbers, may further reflect its lack of proper comprehension and its simultaneous need for the highest level of abstraction.

## Supplementary Information

Below is the link to the electronic supplementary material.

Supplementary file1 (DOCX 128 KB)

## Data Availability

Supplemental materials, stimuli and data associated with this article are available at https://osf.io/kypsr/.
