# Supplementary material for: Symbolic representations of infinity: the impact of notation and numerical syntax
Source: Psychol Res. 2024 Dec 2;89(1):30. doi: 10.1007/s00426-024-02050-8 (PMC11609123; doi:10.1007/s00426-024-02050-8)
Supplement: Supplementary file 1 — Supplementary file1 (DOCX 128 KB) [file 426_2024_2050_MOESM1_ESM.docx]

**Supplementary Materials**

**Word/stimulus length-value congruity effects analyses**

Although the selection of our stimuli was not made based on word/stimulus length-value congruity effects (e.g., Kwon & Oh, 2019; Razpurker-Apfeld & Koriat, 2006; Vaid, 1985), but rather, on the factors of comparison type, distance, and notation, we tested for the significance of these effects in both the verbal and mixed notation blocks. These analyses can provide additional support for the connection between stimuli size/length and numerical value in case significant word/stimulus length-value congruity effects are revealed.

Similar to the word length-value congruity effect expected in the verbal notation block, we hypothesized the congruity between stimulus length and numerical value would influence performance in the mixed notation block. Comparisons in the mixed notation block always involved one symbol (i.e., single digit or infinity symbol) versus a string of symbols (i.e., number word or infinity word). Accordingly, in congruent stimulus length-value trials, the string of symbols was both longer and numerically larger than the one symbol (e.g., nine vs. 3), whereas in incongruent stimulus length-value trials, the string of symbols was longer than the one symbol but numerically smaller (e.g., three vs. 9). Hence, we predicted a word/stimulus length-value congruity effect in the verbal/mixed notation block, demonstrating slower responses for word/stimulus length-value incongruent than congruent trials in the verbal/mixed notation block, respectively.

**Experiment 1**

To examine the influence of word/stimulus length-value congruity in the verbal and mixed notation blocks, respectively, we conducted a GLMM analysis on correct RT with a main effect for word/stimulus length-value congruity (congruent, incongruent) and an interaction between block notation (verbal, mixed) and word/stimulus length-value congruity. A main effect of word/stimulus length-value congruity, χ^2^(1) = 1,485.95, *p* < .001, demonstrated faster responses for word/stimulus length-value congruent (607 ms) than incongruent (717 ms) trials, confirming a word/stimulus length-value congruity effect. Furthermore, the Block Notation × Word/Stimulus Length-Value Congruity interaction was significant, χ^2^(2) = 615.10, *p* < .001 (Fig. S1), revealing a larger word length-value congruity effect (148 ms; verbal notation block), *p* < .001, than a stimulus length-value congruity effect (64 ms; mixed notation block), *p* < .001.

**Fig. S1.** Experiment 1: Estimated mean RTs as a function of block notation and word/stimulus length- value congruity.


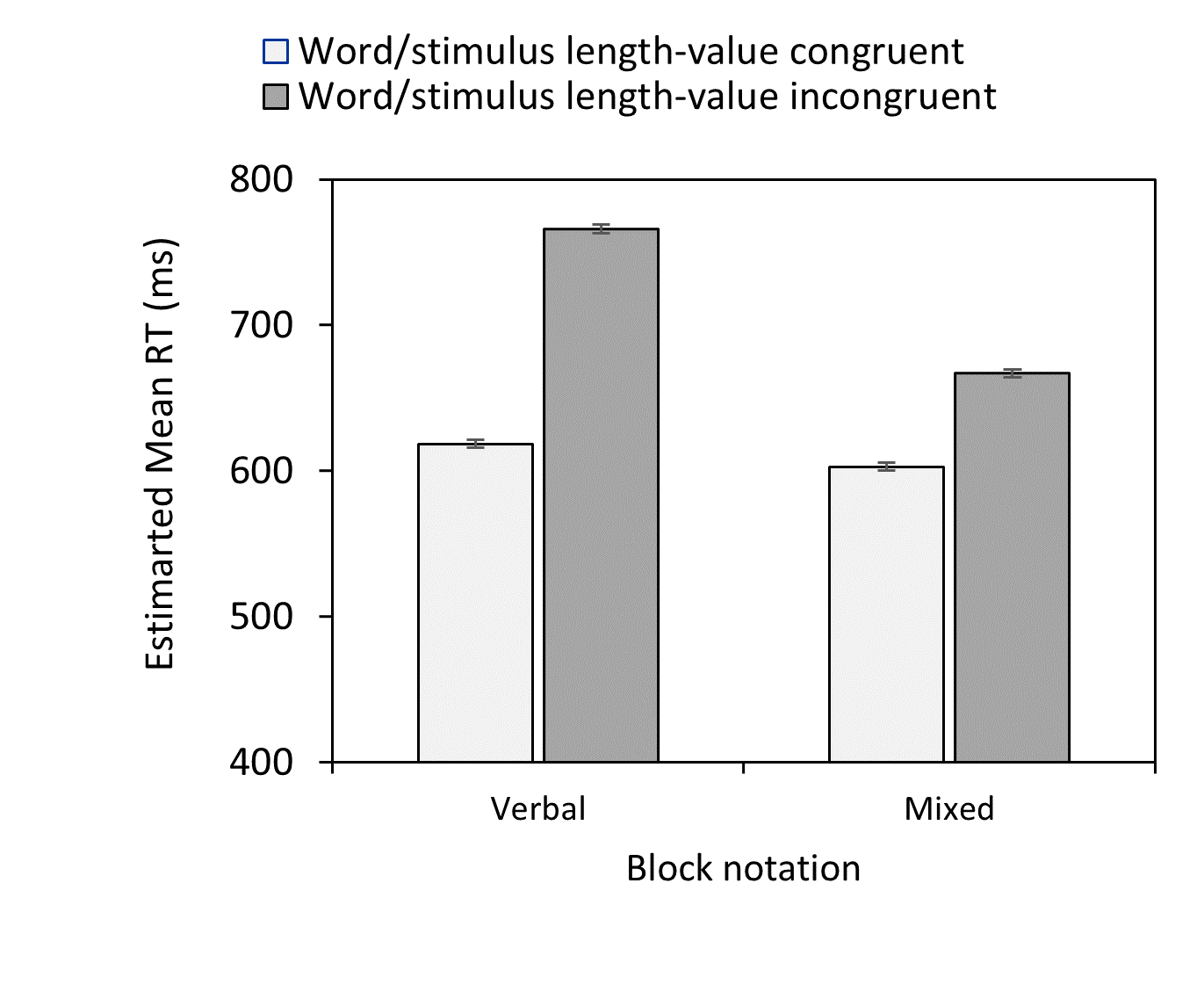


*Note.* Vertical bars denote ± standard errors.

**Experiment 2**

To examine the influence of word/stimulus length-value congruity in the verbal and mixed notation blocks, respectively, we conducted a GLMM analysis on correct RT with a main effect for word/stimulus length-value congruity (congruent, incongruent) and an interaction between block notation (verbal, mixed) and word/stimulus length-value congruity. A main effect of word/stimulus length-value congruity, χ^2^(1) = 10.91, *p* = .001, demonstrated faster responses for word/stimulus length-value congruent (688 ms) than incongruent (709 ms) trials, confirming a word/stimulus length-value congruity effect. Furthermore, the Block Notation × Word/Stimulus Length-Value Congruity interaction was significant, χ^2^(2) = 240, *p* < .001 (Fig. S2), revealing a word length-value congruity effect (98 ms; verbal notation block), *p* < .001, but a reversed stimulus length-value congruity effect (-57 ms; mixed notation block), *p* < .001.

**Fig. S2.** Experiment 2: Estimated mean RTs as a function of block notation and word/stimulus length- value congruity.


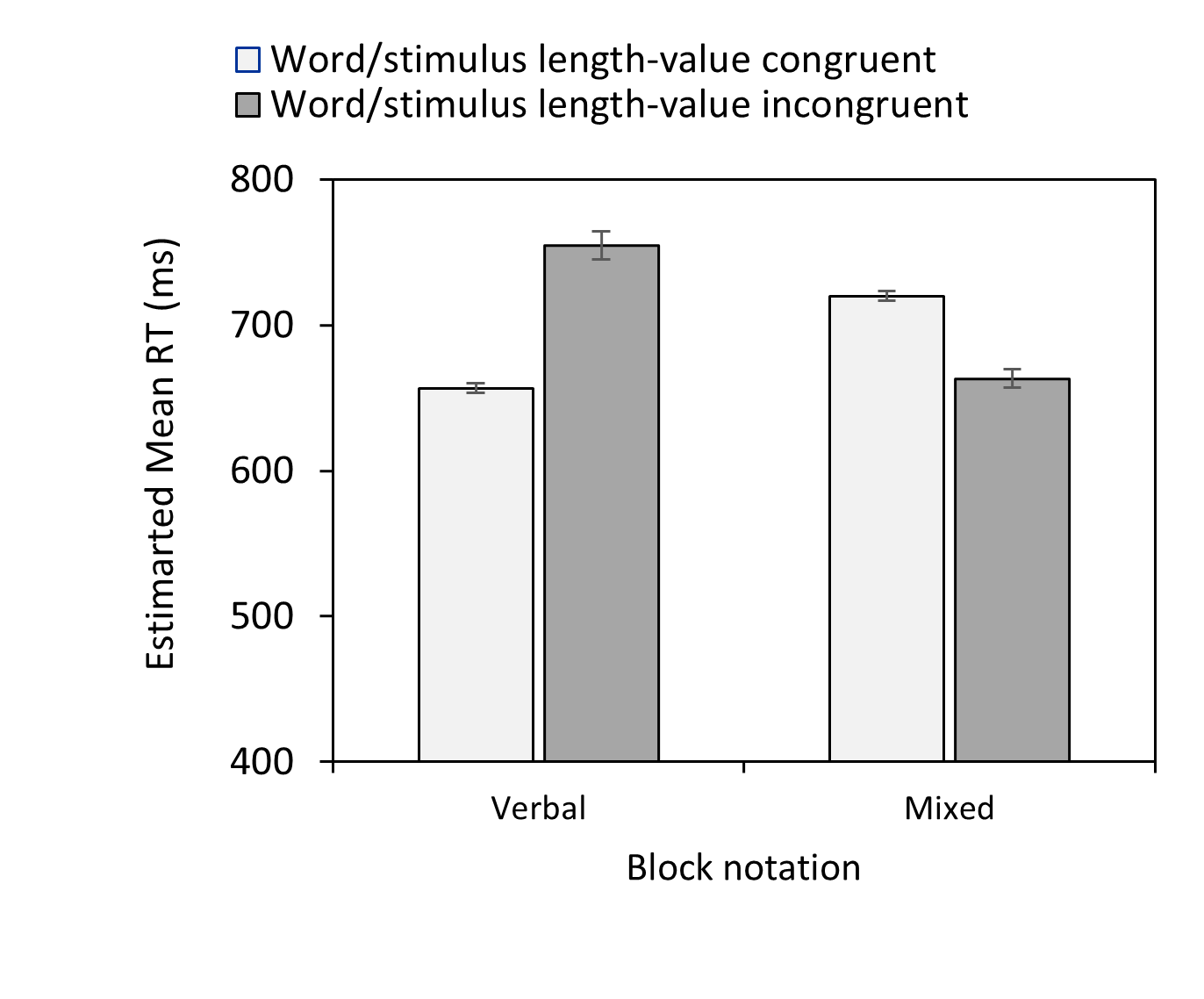


*Note.* Vertical bars denote ± standard errors.

An inconsistency in the findings of the mixed notation block between the experiments was the reversal of the stimulus length-value congruity effect in Experiment 2. This reversed effect may have resulted from the overall greater perceptual variability characterizing the stimulus set in the mixed notation block of Experiment 2 compared to Experiment 1, particularly, the perceptual prominence of the infinity symbol which, in this case, was the only single-symbol stimulus. This resulted in inconsistent relations between the numerical values of the stimuli and their lengths in this block, which presumably led to a reversed stimulus length-value congruity effect. Nevertheless, we obtained a consistent contribution of word length in the verbal notation blocks of both experiments, as was evidenced by significant word length-value congruity effects (e.g., Kwon & Oh, 2019; Razpurker-Apfeld & Koriat, 2006; Vaid, 1985).

**Error rates**

**Table S1.** Estimated mean error rates as a function of block notation, comparison type, and word/stimulus length-value congruity.

| *Experiment* | *Factors* | *Levels* | *M* | *SE* |
| --- | --- | --- | --- | --- |
| Experiment 1 | Notation | Arabic | .03 | .17 |
|  |  | Verbal | .06 | .24 |
|  |  | Mixed | .07 | .25 |
|  | Comparison type | Infinity comparisons | .04 | .19 |
|  |  | Single-digit comparisons | .06 | .24 |
|  | Comparison type (mixed notation) | Infinity symbol vs. number word | .05 | .21 |
|  |  | Infinity word vs. number | .05 | .21 |
|  |  | Number vs. number word | .08 | .27 |
|  | Word/stimulus length-value congruity | Congruent | .03 | .18 |
|  |  | Incongruent | .11 | .31 |
| Experiment 2 | Notation | Arabic | .03 | .17 |
|  |  | Verbal | .05 | .21 |
|  |  | Mixed | .06 | .24 |
|  | Comparison type | Infinity comparisons | .07 | .25 |
|  |  | Multi-digit comparisons | .04 | .19 |
|  | Comparison type (mixed notation) | Infinity symbol vs. number word | .02 | .13 |
|  |  | Infinity word vs. number | .09 | .28 |
|  |  | Number vs. number word | .07 | .25 |
|  | Word/stimulus length-value congruity | Congruent | .03 | .16 |
|  |  | Incongruent | .11 | .31 |

**References**

Kwon, D., & Oh, S. (2019). The number of letters in number words influences the response time in numerical comparison tasks: Evidence using Korean number words. *Attention, Perception, and Psychophysics, 81*(8), 2612–2618. <https://doi.org/10.3758/s13414-019-01870-w>

Razpurker-Apfeld, I., & Koriat, A. (2006). Flexible mental processes in numerical size judgments: The case of Hebrew letters that are used to convey numbers. *Psychonomic Bulletin and Review, 13*(1), 78–83. <https://doi.org/10.3758/BF03193816>

Vaid, J. (1985). Numerical size comparisons in a phonologically transparent script. *Perception and Psychophysics, 37*(6), 592–595. <https://doi.org/10.3758/BF03204927>
